# Clinical profile and contemporary management of patients with heart failure with preserved ejection fraction: results from the CHECK-HF registry

**DOI:** 10.1007/s12471-020-01534-7

**Published:** 2021-01-13

**Authors:** A. Uijl, J. F. Veenis, H. P. Brunner-La Rocca, V. van Empel, G. C. M. Linssen, F. W. Asselbergs, C. van der Lee, L. W. M. Eurlings, H. Kragten, N. Y. Y. Al-Windy, A. van der Spank, S. Koudstaal, J. J. Brugts, A. W. Hoes

**Affiliations:** 1 Julius Centre for Health Sciences and Primary Care, University Medical Centre Utrecht, Utrecht University, Utrecht, The Netherlands; 2 Division of Cardiology, Department of Medicine, Karolinska Institutet, Stockholm, Sweden; 3 Department of Cardiology, Erasmus MC, University Medical Centre Rotterdam, Thoraxcenter, Rotterdam, Rotterdam, The Netherlands; 4 Heart and Vascular Centre, Maastricht University Medical Centre, Maastricht, The Netherlands; 5 Department of Cardiology, Hospital Group Twente, Almelo and Hengelo, Almelo, The Netherlands; 6 Department of Cardiology, Division Heart and Lungs, University Medical Centre Utrecht, Utrecht University, Utrecht, The Netherlands; 7 Institute of Cardiovascular Science and Institute of Health Informatics, Faculty of Population Health Sciences, University College London, London, UK; 8 Streekziekenhuis Koningin Beatrix, Winterswijk, The Netherlands; 9 VieCuri Medisch Centrum, Venlo, The Netherlands; 10 Zuyderland Medisch Centrum, Heerlen, The Netherlands; 11 Gelre Ziekenhuizen, Zutphen, The Netherlands; 12 Flevoziekenhuis, Almere, The Netherlands

**Keywords:** Heart failure with preserved ejection fraction, HFpEF, Comorbidities, Treatment

## Abstract

**Background:**

Clinical management of heart failure with preserved ejection fraction (HFpEF) centres on treating comorbidities and is likely to vary between countries. Thus, to provide insight into the current management of HFpEF, studies from multiple countries are required. We evaluated the clinical profiles and current management of patients with HFpEF in the Netherlands.

**Methods:**

We included 2153 patients with HFpEF (defined as a left ventricular ejection fraction ≥ 50%) from the CHECK-HF registry, which included patients from 2013 to 2016.

**Results:**

Median age was 77 (IQR 15) years, 55% were women and the most frequent comorbidities were hypertension (51%), renal insufficiency (45%) and atrial fibrillation (AF, 38%). Patients between 65 and 80 years and those over 80 years had on average more comorbidities (up to 64% and 74%, respectively, with two or more comorbidities) than patients younger than 65 years (38% with two or more comorbidities, *p*-value < 0.001). Although no specific drugs are available for HFpEF, treating comorbidities is advised. Beta-blockers were most frequently prescribed (78%), followed by loop diuretics (74%), renin-angiotensin system (RAS) inhibitors (67%) and mineralocorticoid receptor antagonists (MRAs, 39%). Strongest predictors for loop-diuretic use were older age, higher New York Heart Association class and AF.

**Conclusion:**

The medical HFpEF profile is determined by the underlying comorbidities, sex and age. Comorbidities are highly prevalent in HFpEF patients, especially in elderly HFpEF patients. Despite the lack of evidence, many HFpEF patients receive regular beta-blockers, RAS inhibitors and MRAs, often for the treatment of comorbidities.

**Supplementary Information:**

The online version of this article (10.1007/s12471-020-01534-7) contains supplementary material, which is available to authorized users.

## What’s new?

- This study provides insight into the medical management of patients with heart failure with preserved ejection fraction (HFpEF) in the Netherlands.
- Additionally, this study demonstrates that the prescription of beta-blockers, renin-angiotensin system inhibitors and mineralocorticoid receptor antagonists in HFpEF patients is primarily determined by age, sex, New York Heart Association (NYHA) class and underlying comorbidities.
- The newly gained insight into the effects of age, sex, NYHA class and comorbidities might aid heart failure specialists in optimising the management of HFpEF.

## Introduction

A large proportion of all heart failure (HF) patients are diagnosed with HF with preserved ejection fraction (HFpEF), with a further increase expected [1–3]. The literature reports an estimated proportion of HFpEF among HF patients of up to 50%, but that percentage is likely to be an underestimation, as many HFpEF patients go unrecognised, especially in primary care [1]. HFpEF is associated with substantial morbidity and mortality, comparable to HF with reduced ejection fraction (HFrEF) [4], with an estimated 1‑year survival after the diagnosis of 78% [5]. HFpEF patients more often have more comorbidities and are older than HFrEF patients [6–8]. So far, there are no evidence-based treatment options for HFpEF patients. Recently, sacubitril-valsartan was not found to have better primary clinical outcomes than valsartan in the treatment of HFpEF [9], despite a large subset of hypertensive patients and a significant blood-pressure-lowering effect. Furthermore, the Swedish Heart Failure registry demonstrated the prognostic impact of non-cardiac comorbidities [6], and European Society of Cardiology (ESC) guidelines currently recommend that only co-existing comorbidities be treated in HFpEF patients [4].

Despite the lack of specific treatment recommendations, many HFpEF patients receive HFrEF medication [10]. However, whether the patient’s clinical profile, such as age and sex, as well as the presence of comorbidities influences the medical management of HFpEF patients remains unclear. With the current analysis of 2153 HFpEF patients in the Dutch registry CHECK-HF (*Chronisch Hartfalen ESC-richtlijn Cardiologische praktijk Kwaliteitsproject-HartFalen*), we aimed to investigate whether the clinical profile and comorbidities influence the contemporary management of HFpEF patients.

## Methods

### Study population

The CHECK-HF is a cross-sectional registry consisting of unselected patients from 34 Dutch hospitals with the diagnosis of chronic HF, according to ESC-guideline definitions, treated at Dutch dedicated outpatient HF clinics (96%) in the period September 2013 to September 2016. The registry comprises 10,910 patients with chronic HF [11, 12] and includes detailed data on baseline characteristics, electrocardiography, echocardiography and laboratory assessments. Details of the design of the registry were published previously [11].

Patients were included if they were 18 years or older and had a diagnosis of HF based on the 2012 ESC guidelines: i.e. structural and/or functional cardiac abnormalities, signs and symptoms of HF [13]. Baseline ejection fraction was assessed by echocardiography. HFpEF was classified as a left ventricular ejection fraction (LVEF) of ≥ 50% with no previously known reduced LVEF. In total, 2267 (21.3%) patients in the registry were classified as HFpEF patients. HFpEF patients in whom no data on drug treatment had been recorded (*n* = 114) were excluded. Therefore, a total of 2153 HFpEF patients were included in this analysis.

This study was conducted in accordance with the Helsinki declaration and was approved by the Medical Ethics Committee 2017 at Maastricht University Medical Centre (Maastricht, the Netherlands).

### Baseline measurements

Baseline variables used in the analyses are described in detail in the design article [11]. For the analysis of comorbidities, we focused on atrial fibrillation (AF), diabetes mellitus (DM), hypertension, hypercholesterolaemia, renal insufficiency (estimated glomerular filtration rate (eGFR) < 60 ml/min or a documented history of renal insufficiency), thyroid dysfunction, peripheral artery disease (PAD), iron deficiency and chronic obstructive pulmonary disease (COPD). AF was defined as a documented history of AF or AF diagnosed by 12-lead electrocardiogram, performed during the most recent outpatient clinic visit.

### Statistical analyses

Baseline continuous variables are presented as mean ± standard deviation or median with interquartile range (IQR) where appropriate; categorical data are presented as numbers and percentages. A chi-square test was used to compare data for categorical variables and a *t*-test or Mann-Whitney U test for continuous data. Additionally, baseline characteristics were analysed in age and sex strata (men < 65 years, men 65–80 years, men > 80 years, women < 65 years, women 65–80 years, and women > 80 years). We investigated the distribution for the number of comorbidities, which was categorised into no comorbidities, one, two, or three or more comorbidities, stratified by age and sex (men < 65 years, men 65–80 years, men > 80 years, women < 65 years, women 65–80 years, and women > 80 years).

Missing data in the baseline measurements (Electronic Supplementary Material, Table S1) were imputed, using multiple imputation, from the mice algorithm in the statistical software package R. Analyses were performed on the ten imputed datasets separately and results were pooled using Rubin’s rules. Multivariable predictors of use of loop diuretics, beta-blockers, renin-angiotensin system (RAS) inhibitors and mineralocorticoid receptor antagonists (MRAs) were assessed using multivariable logistic regression analysis. All predictors of medication use in univariate analysis (data not shown) at a *p*-value of < 0.1 were included, using the enter method, in the multivariable regression analysis. Results are presented as odds ratio and 95% confidence interval. Analyses were performed using SPSS Statistical Package version 25.0 (SPSS Inc., IBM, Armonk, NY, USA) and R version 3.2.3.

## Results

### Baseline characteristics

Baseline characteristics are shown in Tab. 1. Overall, the median age of the HFpEF patients was 77 years (IQR 69–84 years), 54.5% were women and 24.6% had a history of coronary artery disease. Comorbidities were frequently present at baseline, patients had a median of 2 (IQR 1–3) comorbidities, and only 11.4% had no comorbidities. Renal insufficiency (45.3%), hypertension (50.7) and AF (38.4%) occurred most frequently.

**Table 1:** Baseline patient characteristics, stratified by age and sex

				Men			Women			
			Overall	< 65 years	65–80 years	> 80 years	< 65 years	65–80 years	> 80 years	*p*-value
Number			2153	204	452	321	144	453	571	
Age (years), median (IQR)			77 (69–84)	58 (53–61)	72 (69–77)	84 (82–87)	58 (52–62)	74 (70–77)	85 (82–88)	< 0.001
History of coronary artery disease			513 (24.6)	67 (33.2)	171 (38.6)	83 (26.7)	29 (20.9)	84 (19.1)	79 (14.3)	< 0.001
History of cancer			242 (14.1)	10 (5.7)	59 (16.0)	54 (21.1)	17 (13.7)	48 (13.2)	54 (12.4)	< 0.001
History of valvular disease			207 (15.6)	9 (9.8)	45 (16.1)	32 (13.3)	14 (19.4)	55 (20.7)	52 (13.8)	0.067
*Heart failure measures (%)*										
	Ischaemic aetiology HF		612 (29.3)	72 (35.6)	199 (44.9)	108 (34.7)	35 (25.2)	99 (22.5)	99 (17.9)	< 0.001
	NYHA class									
		NYHA I	418 (19.8)	75 (37.9)	122 (27.3)	36 (11.4)	49 (34.3)	75 (16.7)	61 (10.9)	< 0.001
		NYHA II	1038 (49.1)	91 (46.0)	224 (50.1)	173 (54.6)	63 (44.1)	224 (50.0)	263 (47.0)	
		NYHA III	612 (29.0)	30 (15.2)	93 (20.8)	98 (30.9)	31 (21.7)	145 (32.4)	215 (38.4)	
		NYHA IV	45 (2.1)	2 (1.0)	8 (1.8)	10 (3.2)	0 (0.0)	4 (0.9)	21 (3.8)	
	NTproBNP (pmol), median (IQR)		116.8 (40.5–291.5)	30.6 (14.0–178.4)	128.0 (48.4–255.3)	167.0 (55.8–455.8)	77.6 (35.3–389.3)	92.0 (31.4–196.9)	135.1 (42.4–382.1)	< 0.001
*Clinical measurements*										
	BMI (kg/m^2^)		28.4 ± 5.9	29.3 ± 6.4	29.0 ± 5.5	26.8 ± 4.5	29.5 ± 7.6	30.0 ± 6.5	27.3 ± 5.3	< 0.001
	Pulse pressure		62.1 ± 19.2	58.3 ± 14.7	62.7 ± 18.8	60.7 ± 18.3	58.1 ± 18.7	63.7 ± 19.9	63.7 ± 20.7	< 0.001
	DBP (mm Hg)		72.7 ± 12.2	78.9 ± 13.2	73.8 ± 11.1	69.2 ± 11.4	76.7 ± 11.5	72.9 ± 12.4	70.4 ± 12.0	< 0.001
	SBP (mm Hg)		134.8 ± 22.9	137.2 ± 22.2	136.5 ± 22.3	129.9 ± 21.8	134.8 ± 21.6	136.5 ± 23.5	134.2 ± 23.5	< 0.001
	eGFR		61.3 ± 25.3	75.5 ± 21.2	57.7 ± 23.5	49.0 ± 24.7	68.4 ± 21.2	54.6 ± 21.5	45.2 ± 19.4	< 0.001
	Oedema (%)		292 (17.9)	23 (13.5)	58 (16.5)	53 (22.0)	19 (15.6)	54 (15.7)	85 (21.0)	0.086
	Devices (%)		346 (16.1)	31 (15.2)	66 (14.6)	55 (17.1)	17 (11.8)	74 (16.3)	103 (18.0)	0.454
*Comorbidities (%)*										
	Hypertension		1085 (50.6)	90 (44.1)	214 (47.3)	153 (47.7)	66 (45.8)	240 (53.0)	322 (56.4)	0.006
	Diabetes		642 (29.9)	45 (22.1)	159 (35.2)	69 (21.5)	32 (22.2)	183 (40.4)	154 (27.0)	< 0.001
	COPD		109 (19.1)	22 (10.8)	101 (22.3)	79 (24.6)	27 (18.8)	87 (19.2)	93 (16.3)	0.001
	Hypercholesterolaemia		236 (11.0)	28 (13.7)	49 (10.8)	32 (10.0)	17 (11.8)	64 (14.1)	46 (8.1)	0.041
	Renal insufficiency^a^		972 (45.3)	22 (10.8)	164 (36.3)	198 (61.7)	29 (20.1)	203 (44.8)	356 (62.3)	< 0.001
	Atrial fibrillation		817 (38.4)	33 (16.3)	175 (38.9)	152 (47.5)	17 (12.1)	162 (35.9)	278 (49.2)	< 0.001
	Thyroid dysfunction		167 (8.4)	7 (3.7)	18 (4.2)	15 (5.0)	16 (11.9)	51 (12.1)	60 (11.5)	< 0.001
	Peripheral artery disease		71 (3.6)	4 (2.1)	16 (3.8)	13 (4.3)	3 (2.2)	19 (4.5)	16 (3.1)	0.568
	Iron deficiency		11 (0.6)	0 (0.0)	2 (0.5)	1 (0.3)	0 (0.0)	5 (1.2)	3 (0.6)	0.392
	Number of comorbidities (median (IQR))		2 (1–3)	1 (0–2)	2 (1–3)	2 (1–3)	1 (0–2)	2 (1–3)	2 (2–3)	< 0.001

*IQR *interquartile range, *HF* heart failure, *NYHA* New York Heart Association, *NTproBNP N*-terminal pro-B-type natriuretic peptide, *BMI* body mass index, *DBP* diastolic blood pressure, *SBP* systolic blood pressure, *eGFR* estimated glomerular filtration rate, *COPD* chronic obstructive pulmonary disease

^a^Defined as an eGFR < 60 ml/min or a documented history of renal insufficiency

### Distribution of comorbidities

**Fig. 1:**
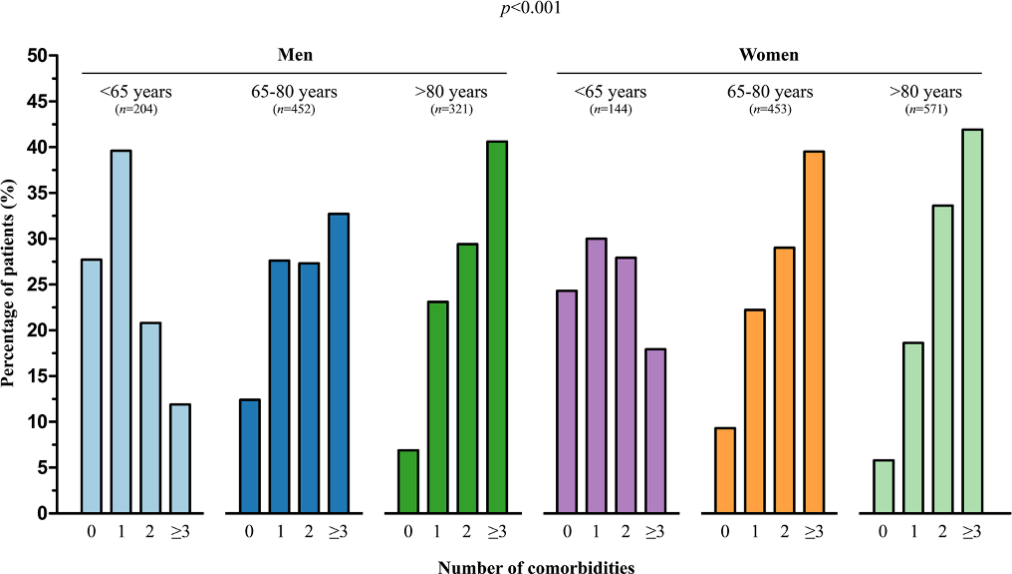
Percentage of patients per number of comorbidities, stratified by age and sex (men < 65 years, men 65–80 years, men > 80 years, women < 65 years, women 65–80 years and women > 80 years)

Fig. 1 shows the distribution for the number of comorbidities ranging from 0 to 3 or more, stratified by age and sex. The younger patients aged < 65 years, both men and women, mainly had 0 or 1 comorbidity, whereas older patients more often had 2 or more comorbidities. Women had 3 or more comorbidities more often than men (*p* = 0.001).

### Medical profile of HFpEF patients

The pharmacological therapy in HFpEF patients is shown in Tab. 2 and is stratified according to age categories, sex, and the presence of hypertension, AF and DM. Loop diuretics were the most frequently prescribed type of HF medication (79.4%), followed by beta-blockers (78.4%), RAS inhibitors (67.3%) and MRAs (38.5%). MRAs, diuretics, digoxin and oral anticoagulants (OACs) were used most often in the oldest age category (*p* < 0.001 for all trends). Diuretics (*p* < 0.001), digoxin (*p* = 0.002) and OACs (*p* < 0.001) were used more often in women than in men. HFpEF patients with hypertension received RAS inhibitors (*p* < 0.001) and diuretics (*p* = 0.016) more often than patients without hypertension. MRAs (*p* < 0.001), diuretics (*p* < 0.001), digoxin (*p* < 0.001), amiodarone (*p* = 0.010), OACs (*p* < 0.001) and non-vitamin K OACs (*p* < 0.001) were prescribed more often to HFpEF patients with AF. Diuretics and statins were prescribed more often to HFpEF patients with DM (*p* < 0.001, for both). MRAs (*p* = 0.005), diuretics (*p* < 0.001) and OACs (*p* = 0.001) were prescribed more often in patients with clinical signs of congestion, while RAS inhibitors were prescribed less often in these patients.

**Table 2:** Profile of medication received by patients with heart failure with preserved ejection fraction

			Diuretics	RAS inhibitor	Beta-blocker	MRA	Digoxin	Amiodarone	OAC	NOAC	Statin
Overall population			1710 (79.4)	1450 (67.3)	1685 (78.3)	828 (38.5)	388 (18.0)	98 (12.8)	1104 (59.2)	79 (4.2)	1754 (81.5)
**Subgroups**											
	*Age*										
		< 65 years	183 (52.4)	240 (68.8)	273 (78.2)	100 (28.7)	40 (11.5)	8 (11.3)	99 (39.3)	9 (3.6)	314 (90.0)
		65–80 years	709 (78.2)	653 (72.0)	720 (79.4)	329 (36.3)	164 (18.1)	51 (17.8)	459 (57.2)	34 (4.2)	786 (86.7)
		> 80 years	816 (91.2)	555 (62.0)	691 (77.2)	399 (44.6)	183 (20.4)	39 (9.6)	545 (67.5)	36 (4.5)	653 (73.0)
		*p*-value	< 0.001	< 0.001	0.534	< 0.001	0.001	0.006	< 0.001	0.831	<0.001
	*Sex*										
		Men	725 (74.2)	658 (67.3)	767 (78.5)	353 (36.1)	148 (15.1)	36 (11.9)	463 (54.4)	32 (3.8)	829 (84.9)
		Women	980 (83.8)	787 (67.3)	915 (78.2)	471 (40.3)	239 (20.4)	61 (13.3)	636 (63.2)	47 (4.7)	920 (78.6)
		*p*-value	< 0.001	0.967	0.866	0.050	0.002	0.568	< 0.001	0.334	< 0.001
	*Hypertension*										
		With HT	890 (81.5)	781 (71.5)	870 (79.7)	406 (37.2)	189 (17.3)	54 (12.7)	580 (61.4)	35 (3.7)	881 (80.7)
		Without HT	820 (77.3)	669 (63.1)	815 (76.8)	422 (39.8)	199 (18.8)	44 (13.0)	524 (57.0)	44 (4.8)	873 (82.3)
		*p*-value	0.016	< 0.001	0.108	0.216	0.382	0.901	0.056	0.245	0.338
	*Atrial fibrillation*										
		With AF	767 (93.3)	543 (66.1)	662 (80.5)	410 (49.9)	293 (35.6)	31 (9.1)	678 (86.3)	51 (6.5)	623 (75.8)
		Without AF	931 (70.9)	899 (68.4)	1,010 (76.9)	410 (31.2)	90 (6.8)	64 (15.3)	415 (39.1)	28 (2.6)	1120 (85.2)
		*p*-value	< 0.001	0.257	0.045	< 0.001	<0.001	0.010	< 0.001	< 0.001	<0.001
	*Diabetes mellitus*										
		With DM	567 (87.9)	445 (69.0)	509 (78.9)	267 (41.4)	123 (19.1)	27 (11.1)	342 (60.6)	18 (3.2)	560 (86.8)
		Without DM	1143 (75.8)	1005 (66.6)	1176 (78.0)	561 (37.2)	265 (17.6)	71 (13.6)	762 (55.7)	61 (4.7)	1194 (79.2)
		*p*-value	< 0.001	0.287	0.632	0.067	0.408	0.323	0.488	0.134	< 0.001
	*Congestion*^a^										
		With congestion	257 (88.0)	173 (59.2)	226 (77.4)	113 (38.7)	53 (18.2)	16 (12.1)	172 (64.4)	7 (2.6)	231 (79.1)
		Without congestion	9689 (72.0)	937 (69.7)	1042 (77.5)	406 (30.2)	217 (16.1)	71 (12.7)	632 (52.9)	54 (4.5)	1059 (78.7)
		*p*-value	< 0.001	0.001	0.978	0.005	0.400	0.851	0.001	0.161	0.887

*RAS* renin-angiotensin system, *MRA* mineralocorticoid receptor antagonist, *OAC* oral anticoagulant, *NOAC* non-vitamin K OAC, *HT* hypertension, *AF* atrial fibrillation, *DM* diabetes mellitus

^a^Indicated by either peripheral oedema or other signs of a hypervolaemic status

**Fig. 2:**
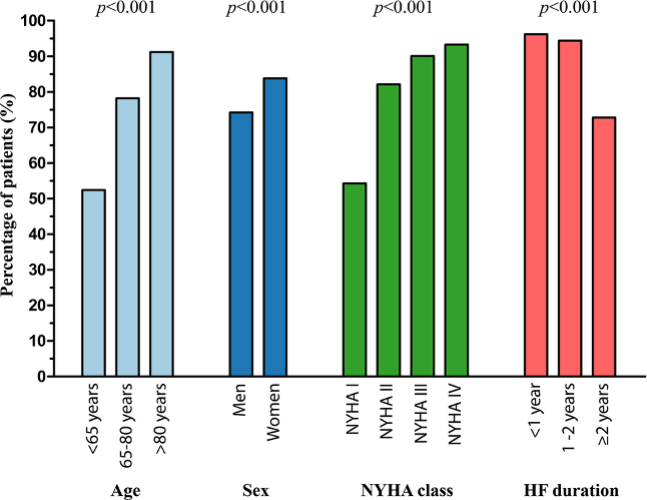
Diuretics profile of patients with heart failure (*HF*) with preserved ejection fraction. *NYHA* New York Heart Association

The distribution of all diuretic use, stratified according to age categories, sex, New York Heart Association (NYHA) class and HF duration is shown in Fig. 2. Diuretics were prescribed more often in older patients, women, patients in a higher NYHA class, and in patients who had been more recently diagnosed with HF (*p* < 0.001).

### Determinants of drug therapy

Independent predictors of the use of loop diuretics, RAS inhibitors, beta-blockers and MRAs are shown in the Electronic Supplementary Material (Figs. S1–S4). Older age, higher NYHA class, higher body mass index (BMI), valvular disease, AF, COPD, DM and concomitant treatment with MRAs and digoxin were all positively associated with loop-diuretic use (Fig. S1) with only higher mean arterial pressure negatively associated with loop-diuretic use. In contrast, lower eGFR and COPD were negatively associated with RAS-inhibitor use (Fig. S2), while hypertension, statin and diuretic use were independent predictors for RAS-inhibitor use. Ischaemic aetiology, higher mean arterial pressure, BMI > 30 kg/m^2^, digoxin and statin use were positively associated with beta-blocker use, while a higher heart rate was a negative predictor (Fig. S3). Lastly, independent predictors for MRA use were: higher NYHA class, lower eGFR, lower mean arterial pressure, AF, valvular disease, PAD, statin and diuretic use (Fig. S4).

## Discussion

In this large contemporary HFpEF cohort, we demonstrated that in daily clinical practice many HFpEF patients receive similar treatment to HFrEF patients, while such treatments are only evidence-based in the latter group [12]. Compared to the HFrEF patient [12], HFpEF patients are older, more often female, and a large proportion of patients have a high number of comorbidities. Pharmacological therapy in HFpEF patients is primarily determined by age, sex, NYHA class and underlying comorbidities, such as renal insufficiency, AF and hypertension.

### HFpEF and comorbidities

The CHECK-HF registry included a large number of elderly persons and a high percentage of women, with many comorbidities, a patient population comparable with current practice in other Western European countries [8, 10, 14]. As in previous reports, AF, renal insufficiency, diabetes and hypertension are the most common reported comorbidities in HFpEF patients [6, 15, 16]. Our results confirm that comorbidities are more prevalent with increasing age [17].

Clarification of the distribution of comorbidities in HFpEF patients is important, since it has been shown that HFpEF patients could be differentiated into several subgroups, based on comorbidities and other clinical parameters [18]. It has been shown that these HFpEF subgroups have significant differences in HF prognosis [18]. Some beneficial effects of treatments recommended for HFpEF patients have been demonstrated in specific HFpEF subgroups, suggesting that an HFpEF phenotype-specific treatment strategy may be warranted [19].

### Drug therapy prescribed to HFpEF patients

Despite the lack of guideline-recommended treatment for HFpEF patients [4], the prescription rates of beta-blockers and RAS inhibitors were high in the CHECK-HF registry, similar to other European cohorts [8, 10, 14]. These medications were most likely prescribed for the treatment of comorbidities, such as hypertension and AF. Similarly, many HFpEF patients received loop diuretics, which were most likely prescribed to treat congestion, as recommended by the HF guidelines [4]. Multivariable analysis showed that the most important determinants of the medication profile are the presence of hypertension, congestion and a higher NYHA class.

The results from the Swedish Heart Failure Registry, demonstrating a reduced all-cause mortality in HFpEF patients treated with beta-blockers compared with patients without beta-blockers, might have influenced physicians in prescribing beta-blockers in HFpEF patients. [20]. Additionally, a recent Cochrane review, including 1046 patients from three randomised controlled trials, demonstrated a significant reduction in all-cause mortality, but no reduction in HF-related hospitalisations [21], although the findings of the Cochrane review could not have influenced our results.

Hypothetically, physicians might have been influenced to prescribe MRAs to reduce left ventricular remodelling and fibrosis in HFpEF patients, as a recent Cochrane review demonstrated a beneficial effect of MRAs in preventing HF hospitalisations in HFpEF patients [21]. Furthermore, a post hoc analysis of the TOPCAT trial, investigating spironolactone, showed regional differences between the Americas and Russia/Georgia, indicating that MRAs might have beneficial effects on mortality in the former [22]. Randomised trials investigating the effects of RAS inhibitors in HFpEF patients did not show a reduction in mortality or HF-related hospitalisations [21]. Most of these trials were underpowered or could have been biased due to the large heterogeneity of the HFpEF population. In contrast, some observational studies have demonstrated an association between RAS-inhibitor use and lower all-cause mortality in HFpEF patients [23]. Importantly some of the HF drugs may have been prescribed simply because patients were diagnosed with HF (in this case HFpEF) and because physicians (and possibly also their patients) felt that the prescription of medication may confer prognostic benefit.

### Strengths and limitations

This study has several strengths. First, the CHECK-HF registry is currently one of the largest European heart failure registries. Another strength is the detailed information on medication use and comorbidities. Third, this cohort included a large subset of HFpEF patients with a diagnosis according to ESC guidelines. A limitation of this study is the lack of follow-up data. Therefore, no associations can be studied for clinical outcomes or mortality. In addition, specific reasons for prescribing medication were not recorded; therefore, any conclusions remain speculative. Finally, in a considerable number of patients, data on eGFR were missing. Although multiple imputation was used to adjust for the missing values, some bias might have occurred.

### Conclusion

We demonstrated that many of the 2153 HFpEF patients in this large contemporary cohort receive beta-blockers, RAS inhibitors and MRAs. The prescription of beta-blockers, RAS inhibitors and MRAs in HFpEF patients is primarily determined by age, sex, NYHA class and underlying comorbidities.

## Supplementary Information

Determinants of heart failure medication usage in HFpEF patients
