# Supplementary material for: Clinical profile and contemporary management of patients with heart failure with preserved ejection fraction: results from the CHECK-HF registry
Source: Neth Heart J. 2021 Jan 13;29(7-8):370–6. doi: 10.1007/s12471-020-01534-7 (PMC8271056; doi:10.1007/s12471-020-01534-7)

**Supplementary material:**

| **Table S1.** Missing baseline patient characteristics | | |
| --- | --- | --- |
|  | **N missing** | **% Missing** |
| **Demographics** |  |  |
| Age | 2 | 0.1 |
| Sex | 6 | 0.3 |
| **Heart failure measures** |  |  |
| NYHA | 34 | 1.6 |
| Devices | 0 | 0 |
| **Clinical measurements** |  |  |
| BMI | 167 | 7.8 |
| Heart rate | 18 | 0.8 |
| MAP | 13 | 0.6 |
| eGFR | 892 | 41.4 |
| **Comorbidities** |  |  |
| Ischaemic heart disease | 0 | 0 |
| Valvular disease | 0 | 0 |
| Hypertension | 0 | 0 |
| Diabetes | 0 | 0 |
| COPD | 0 | 0 |
| Atrial fibrillation | 0 | 0 |
| Peripheral artery disease | 0 | 0 |
| Anaemia | 0 | 0 |
| Iron deficiency | 0 | 0 |
| Thyroid disease | 0 | 0 |
| **Medication use** |  |  |
| Loop diuretics | 0 | 0 |
| RAS inhibitors | 0 | 0 |
| Beta-blockers | 0 | 0 |
| MRA | 0 | 0 |
| NYHA: New York Heart Association; BMI: Body Mass Index; MAP: Mean Arterial Pressure; eGFR: estimated Glomerular Filtration Rate; COPD: Chronic Obstructive Pulmonary Disease; RAS inhibitors: Renin-Angiotensin System Inhibitors; MRA: Mineralocorticoid Receptor Antagonists | | |

**Fig. S1.** Determinants of diuretic use in HFpEF patients.


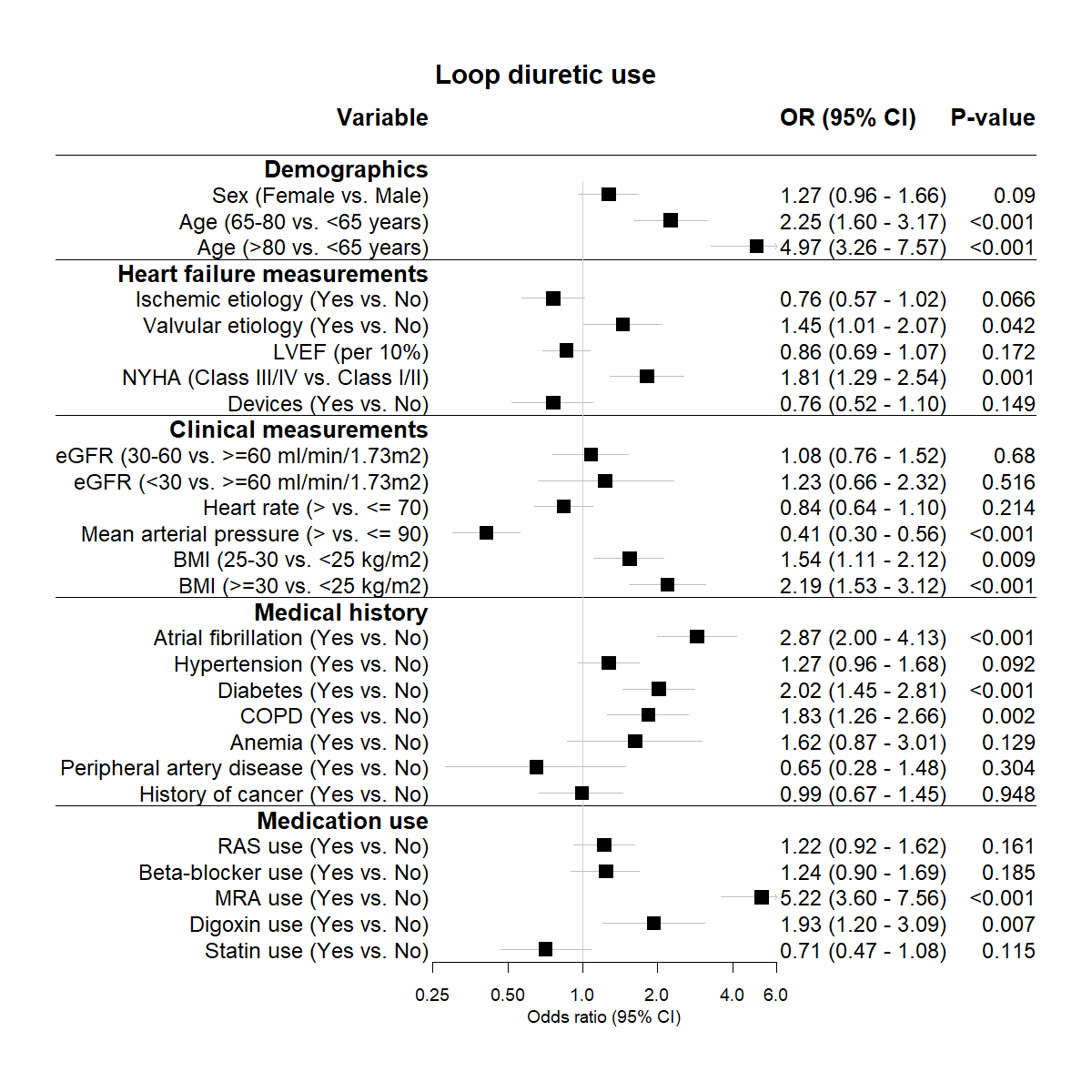


**Fig. S2.** Determinants of RAS-inhibitor use in HFpEF patients.


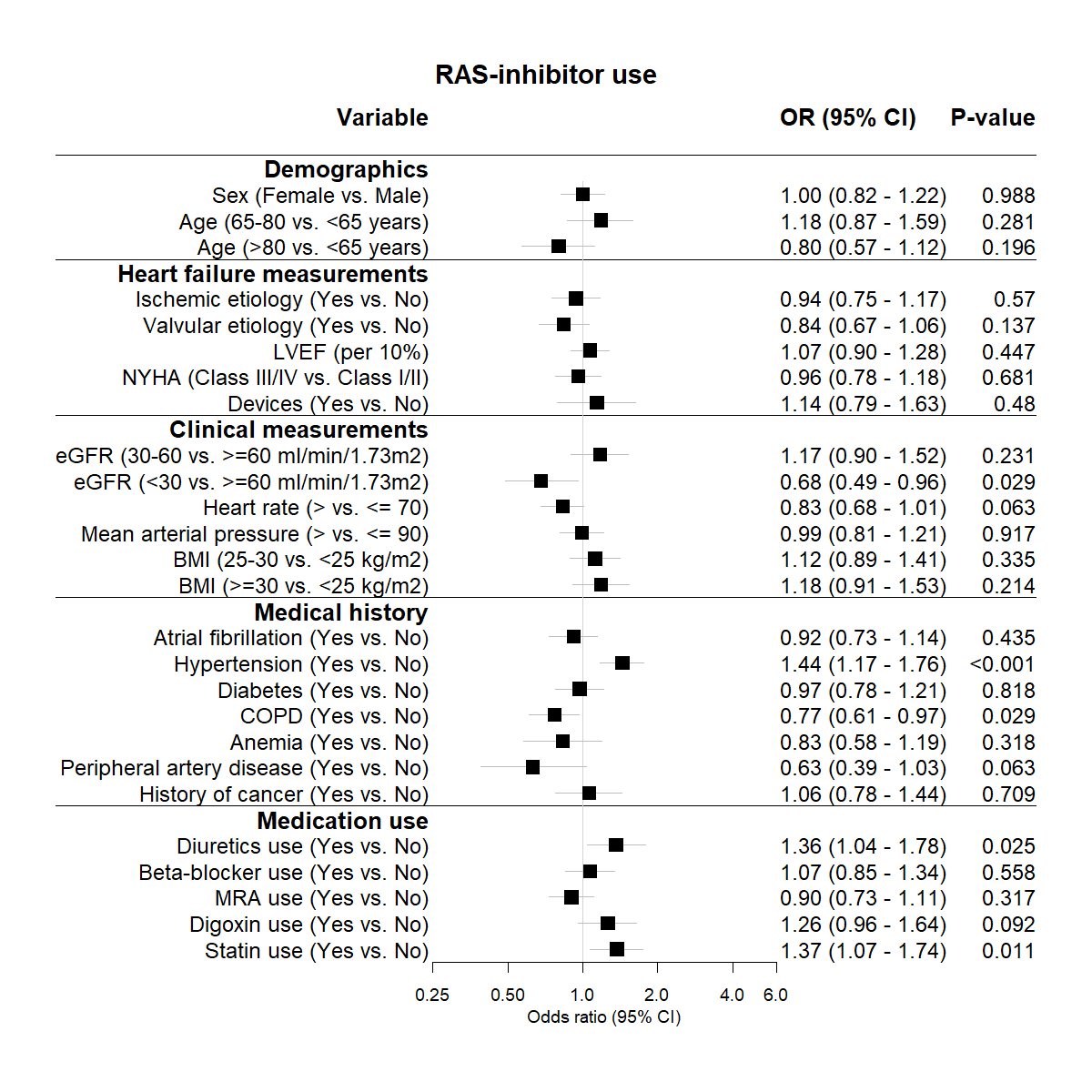


**Fig. S3.** Determinants of beta-blocker use in HFpEF patients.


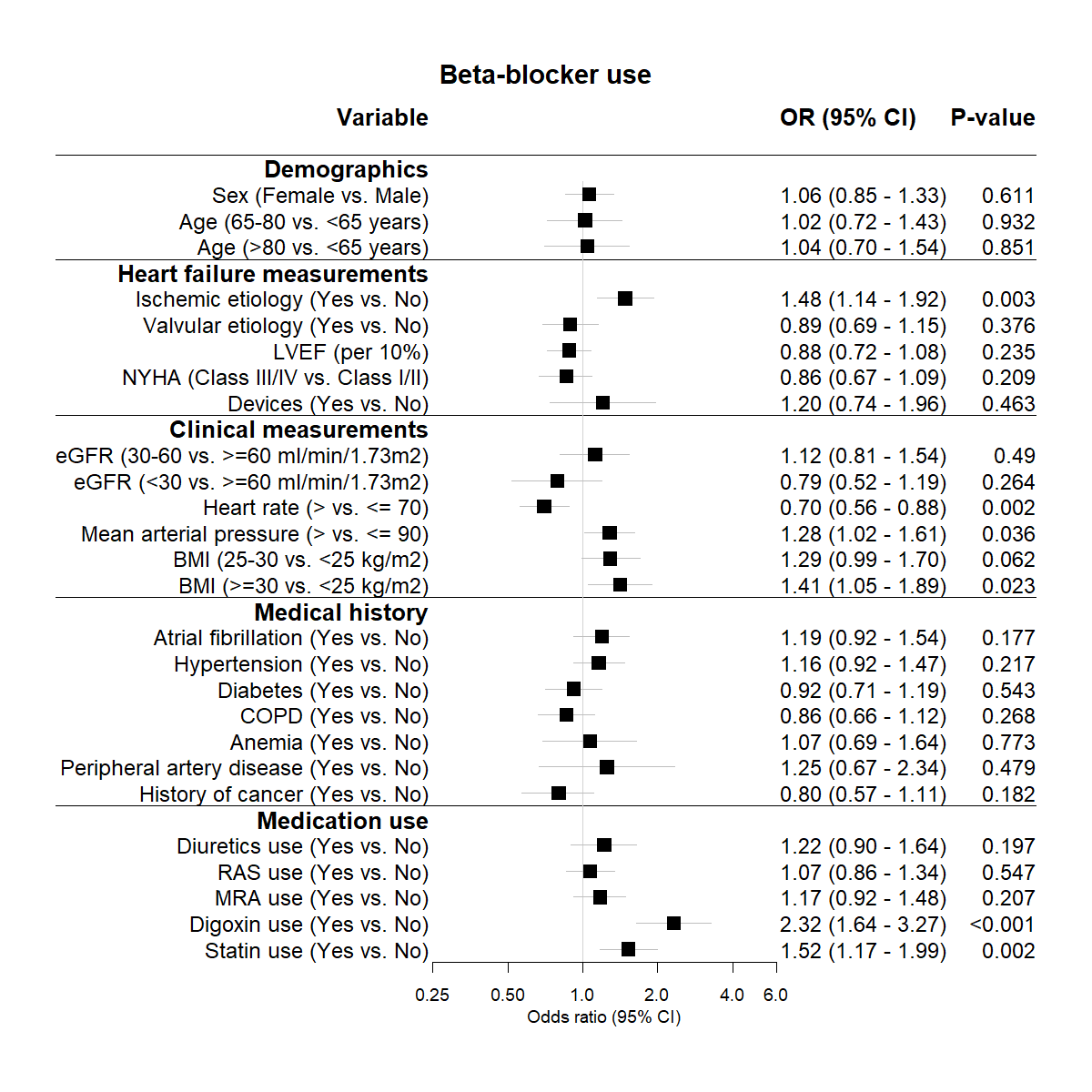


**Fig. S4.** Determinants of MRA use in HFpEF patients.


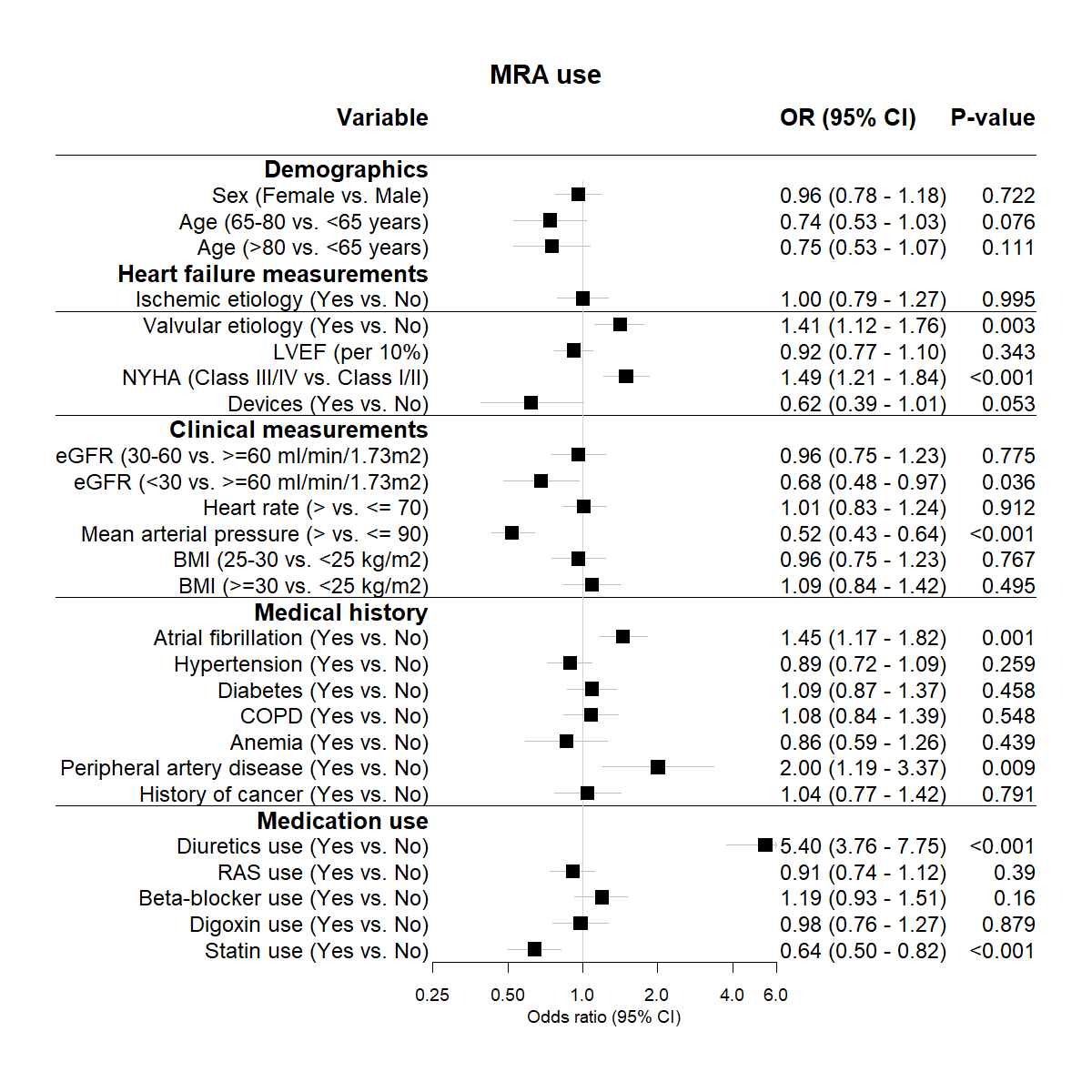

Supplement: Supplementary file 1 — Determinants of heart failure medication usage in HFpEF patients [file 12471_2020_1534_MOESM1_ESM.docx]
